# Factors Impacting Readiness to Perform Secondary Population-Based Triage During the Second Wave of COVID-19 in Victoria, Australia: Pilot Study

**DOI:** 10.1017/dmp.2023.41

**Published:** 2023-03-09

**Authors:** Zachary B. Horn

**Affiliations:** School of Medical and Health Sciences, Edith Cowan University, Western Australia and School of Medicine and Dentistry, Griffith University, Queensland, Australia

**Keywords:** COVID-19, crisis resource management, disaster planning, population-based triage, resource allocation, ICU, intensive care unit, S-PBT, secondary population-based triage

## Abstract

**Objective:**

Pandemics generate such a significant demand for care that traditional triage methods can become saturated. Secondary population-based triage (S-PBT) overcomes this limitation. Although the coronavirus disease (COVID-19) pandemic forced S-PBT into operation internationally during the first year of the pandemic, Australian doctors were spared this responsibility. However, the second wave of COVID-19 provides an opportunity to explore the lived experience of preparing for S-PBT within the Australian context.

The aim of this study is to explore the lived experience of preparing to operationalize S-PBT to allocate critical care resources during Australia’s second wave of COVID-19 in 2020.

**Methods:**

Intensivists and emergency physicians working during the second Victorian COVID-19 surge were recruited by purposive non-random sampling. Semi-structured interviews were hosted remotely, recorded, transcribed, and coded to facilitate a qualitative phenomenological analysis.

**Results:**

Six interviews were conducted with an equal mix of intensivists and emergency doctors. Preliminary findings from a thematic analysis revealed 4 themes: (1) threat of resources running; (2) informed decision requiring information; (3) making decisions as we always do; and (4) a great burden to carry.

**Conclusion:**

This is the first description of this novel phenomenon within Australia and, in doing so, it identified a lack of preparedness to operationalize S-PBT during the second wave of COVID-19 in Australia.

During catastrophic surges, such as pandemics, traditional triage can prioritize so many patients that it fails to guide resource allocation.^1,2^ Secondary population-based triage (S-PBT) can overcome this saturation by considering the population context and overall service availability.^1–4^

Intensive care units (ICUs) are particularly vulnerable during surges due to the resource-intensive care they deliver.^5^ In Australia, ICUs operate under the “closed unit” model with care directed and managed by intensivists. Recent studies identified that intensivist decision-making is influenced by patient, physician, and environmental factors.^6,7^ Unit capacity and bed availability are important factors, but, during business-as-usual, modifying other factors are considered rather than driving decision-making^8,9^; however, their prominence in ICU governance decisions increases during critical surges, representing a transition to S-PBT.

Despite a few proposed, but unvalidated, protocols, there is significant sparsity in literature around S-PBT. No literature exploring the lived experience of S-PBT has been identified, until recently, as this concept had not been widely and systematically operationalized. However, within the first year of the COVID-19 pandemic, international health systems faced such overwhelming demand that S-PBT operationalization became either operationalized or threatened.

Although system strain was acutely felt in Melbourne, Australia, Australian health systems experienced significantly less demand during the second wave compared to international counterparts, and thus S-PBT was not systematically operationalized in Australia. The experience of Australian doctors preparing for S-PBT operationalization may assist in identifying gaps in preparedness and predicting possible outcomes had this phenomenon manifested. This pilot research therefore aimed to explore the lived experience of doctors preparing to operationalize S-PBT during Australia’s second wave of COVID-19 in 2020.

## Methods

Emergency physicians and intensivists working in Victoria during the second surge of COVID-19 in 2020 were purposively sampled. Invitations were sent to publicly prominent clinicians, identified either by presence in public mass media or literature. Semi-structured interviews were conducted via Zoom^TM^, lasted approximately 30 minutes, and utilized interview guides. Interviews were audio-recorded and were manually transcribed.

Data analysis utilized Colaizzi’s approach,^10^ allowing researchers to describe participant experiences and identify emerging themes and relationships. Relevant statements were extracted and summarized by considering content and context (open coding), thus codes were derived de novo. Formulated meanings were grouped (axial coding) to reveal categories in the voice of research participants, and overarching themes were used to formulate a phenomenological description.

Ethical approval was granted by the Edith Cowan University Human Research Ethics Committee (Joondalup, Western Australia). Reference: 2021-02477-HORN.

## Results

Approximately 65 invitations were sent but confirmation of receipt was not sought. Six participants were interviewed with an equal mix of specialties. All participants were male. One additional participant withdrew from the study due to workload in the context of another surge in COVID-19 cases. Several invitation replies cited increasing workload as preventing commitment to an interview.

Thematic analysis of the conducted interviews revealed 4 themes, each with several subthemes (Table 1).

**Table 1.** Key themes and subthemes

Themes	Subthemes
The threat of running out of resources	Concerns arising from the experience of others
	Surging to avoid a crisis
An informed decision required information	Understanding the prognosis
	Respecting patient wishes
	Accurate awareness of capacity
A great burden to carry	The personal and professional burden
	Hospitals and governments can lighten the load
	Carrying the burden with colleagues and the profession
Making decisions as we always do	Practical and effective decision-making
	Decisions made by those who are most experienced

### Threat of Running Out of Resources

Early concerns around the S-PBT arose almost exclusively from international experiences before system strain occurred in Australia:

*… we were looking at the experience in northern Italy … the way they were having to deal with that was pretty horrifying from our perspective…* (P5)

Early experiences threatened confidence in surge capacity; however, some interviewed doctors became so confident in surge capacity that there was resistance to consider the possibility of S-PBT operationalization due to equipment shortages:

*You hear the … horror stories of hospitals running out of ventilators… [but] for instance, in Bergner when they were running out of ventilators, there’s a whole load of ventilators down the road in Rome – so if you have the ability to match demand to capacity, you take away the requirement for needing triage-based decisions…* (P3)

*… each of the other variables can be manipulated, you know, by admitting patients earlier, by admitting patients later, moving patients around the state, moving patients to other states – there’s so many ways it can be manipulated with the right resourcing…* (P2)

### Informed Decision Requiring Information

Prognostic uncertainty threatened clinician readiness to perform S-PBT and was a recurrent theme across interviews. This also complicated managing patient expectations around the appropriateness of ICU care, particularly when already concerned about health literacy among the general population:

*Many people [were] expecting that we’re going to make them better and send them home. And so that’s a really big challenge with a new disease like COVID, [when] we don’t even completely understand the long-term implications of what’s going on.* (P5)

*You can talk about resource rationalization and access to ICU, and most people nod their head … but when it really comes down to the crunch … and what this actually means, I’m not even sure people really understand that ‘limitation of treatment’ concept. (P5)*

Doctors believed denying care that was futile would feel business-as-usual, but they remained concerned about resource-driven decisions which conflict with patient prognoses:

*I think the challenge is probably more likely to be knowing you’ve got otherwise well middle-aged people, … who ordinarily would expect to be sick, go [to ICU], survive, [and] go home who may be dying because they can’t access the resources they need.* (P5)

Accurate and timely awareness of health system capacity, including surveillance and feedback, was critical in the readiness of doctors to operationalize S-PBT:

*The problem I have is that … [we] are making decisions that are different from normal because of perceptions about resource constraints which may not be there. … it’s more about calling out what the reality of the situation is, not being caught up on the concern and perception that may not be as bad as people think.* (P4)

*I think there is a requirement to have scrutiny over … the impact of the decision-making …knowing that systems are watching what the outcomes of those decisions are makes [for] better decisions… (P3)*

### Great Burden to Carry

While participants felt they would be able to perform S-PBT decision-making, they did not expect to remain unaffected:

*… it would have had an impact on subsequent reflection and rumination, and the soul-searching that would no doubt follow.* (P1)

*It would be emotionally draining if that becomes sustained … I think that ends careers. There’s people that won’t get over that sort of experience… (P5)*

Doctors expected hospitals and governments to ensure resources were managed to avoid S-PBT and that, if it came to it, that they would support the decisions of their doctors and protect them from criticism and consequences:

*… it’s really essential for clinicians to do this work … and that they do so knowing that, as long as they had practiced within the guideline’s recommendations and within what was reasonable and proportionate to the circumstances, that we have the backing of our hospital executives and departments of health…* (P1)

*I would like to know that I and my colleagues would be supported in the decision-making process … whether it be by our critical care colleagues or ultimately … by the hospital executive or the department of health and government. (P4)*

Significant support and reassurance came from acknowledgment within the health care community that S-PBT may become required:

*… there was a recognition amongst our peer clinicians … that it might for the first time in our professional lives come to a point whereby considerations beyond patient needs might determine our treatment pathways. … it would have felt very uncomfortable indeed if the reality was we had to do it but nobody else felt or really understood the need.* (P1)

## Making Decisions as We Always Do

Doctors felt that they should remain responsible for allocating resources they routinely manage:

*If we are talking about critically ill patients that need ICU … ICU doctors need to be involved in that decision-making … that’s ICU bread and butter. … [When] we are talking about emergency doctors making the decision as to whether people are admitted to hospital … that is their domain to make those decisions anyway – that’s exactly what they do, that is their everyday…* (P3)

*Emergency physicians have always had a ‘systems view’ … how the hospital works is really important to you because it impacts on patient flow, getting patients in through the system, et cetera. I think we come with … knowledge of the system that potentially others … may not have. (P6)*

Doctors were preparing to make S-PBT decisions individually, reporting that deferring to a panel felt like an impractical interruption to clinical care and that critical care resource allocation is too complex for scoring systems to be an acceptable alternative to clinical assessments:

*It’s actually really difficult to have [triage committees] because … when you actually walk up to a critically ill patient, you need to make the decision there and then – you can’t go to your committee … so, in most instances they’re impractical.* (P3)

*The problem is that it’s just almost impossible to really come up with hard objective criteria because there are just so many variables that come into play. (P2)*

## Discussion

This pilot study sought to explore factors that impacted the readiness of doctors to operationalize S-PBT during the second wave of COVID-19 in Victoria, Australia, in 2020. It is critical to acknowledge that “readiness” was explored in the context of preparing to operationalize S-PBT as it did not become necessitated during that period.

Preliminary findings support and reinforce recently explored ICU admission decision-making factors (patient, clinician, and environmental factors)^8,9^ and suggest that clinician readiness to operationalize S-PBT was heavily influenced when such factors were threatened by the pandemic. This is supported by prognostic uncertainty and barriers to understanding patient wishes reducing clinician readiness by directly threatening the ability of clinicians to integrate routine patient factors in S-PBT decision-making.

System capacity and bed availability, important environmental factors in standard ICU decision-making, become critical drivers during S-PBT operationalisation.^9^ International experiences of the pandemic demonstrated that surge capacity could become overwhelmed, producing an initial crisis-of-faith among interviewed participants. However, Australia’s experience of the second wave seemed to instill such significant confidence in local surge capacity that some interviewed clinicians became unable, or unwilling, to consider future resource shortages necessitating S-PBT operationalization.

Several participants emphasized that they were not ready to implement score-based triage systems, preferring to weigh factors as they usually do; however, this had not been tested at the time of interviews. This also highlights conflicts between government policies and plans, which preference scoring systems, and frontline clinician expectations.

Finally, participants noted that initial concerns drove the sporadic and uncoordinated development of guidelines and policies at the local or even departmental level rather than a coordinated approach so central policies can be contextualized locally. In addition to threatening clinical readiness, this highlights that interviewed clinicians were impacted by a lack of policy and guidance to inform S-PBT operationalization.

### Recommendations

Further research is required and should consider multi-disciplinary experiences of a larger number and scope, temporally and geographically, of participants; this methodology has demonstrated its feasibility. Although grounded in only preliminary findings, governments and health systems should aim to enhance system capacity surveillance and reporting to assist clinician decision-makers. Frontline clinician input should be increased in plans that establish the environment in which S-PBT decisions may arise. Finally, clinicians should be provided explicit reassurance and protection from consequences following S-PBT operationalization.

### Limitations

This study utilized purposive recruitment, a common and accepted practice in qualitative research, and utilized a very small sample. Result transferability is limited by the influence of policy and political landscapes within each Australian jurisdiction. Caution should be exercised when attempting to transfer findings grounded in policy and jurisdictional environments specific to this study.

## Conclusion

These preliminary findings provide the first description of this novel phenomenon in Australia. Several gaps in preparedness were identified, including conflict between policies and stakeholder expectations, deficits in accurate and timely information, and deficits in clinician support and protection. This insight into the lived experience of Victorian doctors ultimately suggested that health systems and clinicians were not prepared to operationalize S-PBT in response to COVID-19; however, additional research is required and should consider this methodology to explore subsequent local and international experiences to further understand this phenomenon.
